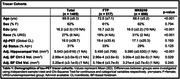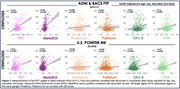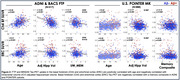# Quantification of tau pathology in the basal forebrain: Associations with age, amyloid, brain structure and cognition

**DOI:** 10.1002/alz.091080

**Published:** 2025-01-09

**Authors:** Trevor Chadwick, Tyler J. Ward, Suzanne L. Baker, William J. Jagust, Susan M. Landau, Theresa M. Harrison, the U.S. POINTER Study Group, the Alzheimer's Disease Neuroimaging Initiative

**Affiliations:** ^1^ University of California, Berkeley, Berkeley, CA USA; ^2^ Lawrence Berkeley National Laboratory, Berkeley, CA USA

## Abstract

**Background:**

Tau pathology accumulates early in the basal forebrain (BF) in Alzheimer’s disease (AD). The feasibility of measuring in vivo BF tau is unclear given PET resolution and possible partial volume effects of off‐target signal (OTS) which varies by tracer.

**Method:**

We compared measurements of tau in cognitively unimpaired older adults with either an FTP or MK6240 scan: 93 FTP scans from the Berkeley Aging Cohort Study (BACS), 424 FTP scans from ADNI (N=517 FTP scans; 72.5±7.1 years, 61% F) and 888 MK6240 scans (68.4±5.2 years, 62% F) from the U.S. POINTER imaging study were processed identically to derive SUVRs in native space FreeSurfer regions and template space BF. Using linear regression we explored associations between tau‐PET ROIs across the brain focusing on the temporal lobe (entorhinal cortex [ERC], temporal MetaROI), BF (Ch1‐3, Ch4) and common regions of OTS (e.g., pallidum, thalamus). We examined relationships between tau in these ROIs and age, Aβ, hippocampal volume, BF volume, and cognition.

**Result:**

The MK6240 cohort was significantly younger, less educated, more racially diverse, and had higher hippocampal and BF Ch1‐3 volume (Table. 1). For both tracers, BF and temporal lobe ROI SUVRs were positively correlated with Aβ, while OTS ROI SUVRs were not related to Aβ (Figure 1). In the BF, the effect size (Cohen’s d) of t‐tests comparing tau SUVR by Aβ status was higher using MK6240 compared to FTP. BF and ERC SUVR positively correlated with age and negatively correlated with hippocampal volume and episodic memory (Figure 2). BF Ch4 SUVR negatively correlated with BF Ch4 volume in both cohorts.

**Conclusion:**

Evidence that BF tau, along with temporal lobe tau, correlates with Aβ uptake suggests that AD‐relevant tau pathology can be measured in vivo in the BF. In addition, BF tau and temporal tau showed similar relationships to other key variables including age, brain structure and cognition with both tracers. MK6240 slightly better differentiated Aβ positive and negative subjects in BF SUVR measurements compared to FTP. Future research will investigate if BF tau is a critical early marker of AD progression.